# The mitochondrial gene-CMPK2 functions as a rheostat for macrophage homeostasis

**DOI:** 10.3389/fimmu.2022.935710

**Published:** 2022-11-14

**Authors:** Prabhakar Arumugam, Meghna Chauhan, Thejaswitha Rajeev, Rahul Chakraborty, Kanika Bisht, Mahima Madan, Deepthi Shankaran, Sivaprakash Ramalingam, Sheetal Gandotra, Vivek Rao

**Affiliations:** ^1^ Immunology and Infectious Disease Unit, Council of Scientific and Industrial Research (CSIR)- Institute of Genomics and Integrative Biology, New Delhi, India; ^2^ Academy of Scientific and Innovative Research (AcSIR), Council of Scientific and Industrial Research (CSIR)- Human Resource Development Centre, Ghaziabad, India; ^3^ Genomics and Molecular Medicine, Council of Scientific and Industrial Research (CSIR)- Institute of Genomics and Integrative Biology, New Delhi, India

**Keywords:** CMPK2, M1 macrophage, immuno-metabolism, mitochondria, infection

## Abstract

In addition to their role in cellular energy production, mitochondria are increasingly recognized as regulators of the innate immune response of phagocytes. Here, we demonstrate that altering expression levels of the mitochondria-associated enzyme, cytidine monophosphate kinase 2 (CMPK2), disrupts mitochondrial physiology and significantly deregulates the resting immune homeostasis of macrophages. Both CMPK2 silenced and constitutively overexpressing macrophage lines portray mitochondrial stress with marked depolarization of their membrane potential, enhanced reactive oxygen species (ROS), and disturbed architecture culminating in the enhanced expression of the pro-inflammatory genes IL1β, TNFα, and IL8. Interestingly, the long-term modulation of CMPK2 expression resulted in an increased glycolytic flux of macrophages akin to the altered physiological state of activated M1 macrophages. While infection-induced inflammation for restricting pathogens is regulated, our observation of a total dysregulation of basal inflammation by bidirectional alteration of CMPK2 expression only highlights the critical role of this gene in mitochondria-mediated control of inflammation.

## Graphical Abstract

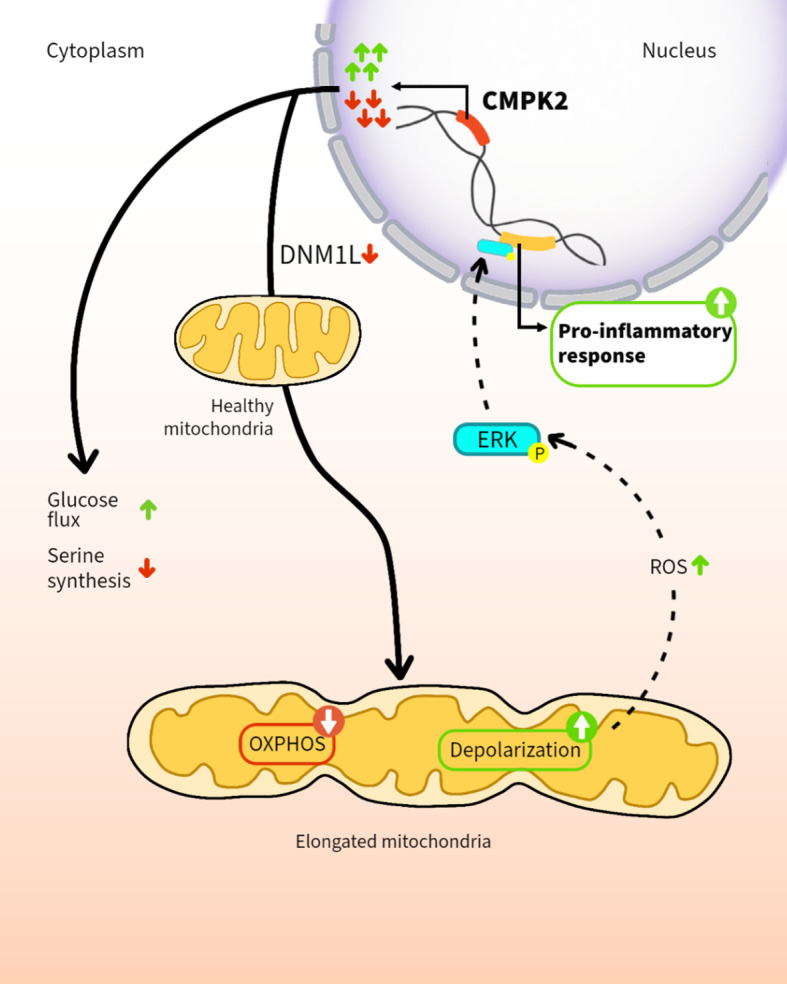

## Introduction

Intracellular microbial infections compel host macrophages to activate selective cellular pathways that are targeted toward the elimination of the pathogen. In response to bacterial infections like *Mycobacterium tuberculosis* (Mtb), in addition to the expression of pro-inflammatory response mediators that facilitate the activation of bactericidal pathways (M1 polarized), recent evidence has demonstrated the robust activation of type I interferon (IFN) response in macrophages (1–4). While the importance of the individual genes of this response cascade (interferon-stimulated genes (ISG)) in restricting viral nucleic acids is well recognized, its contribution to controlling bacterial infections is still unclear (5–7).

One of the early and highly expressed ISGs in macrophages following infection is the mitochondria-associated kinase cytidine monophosphate kinase 2 (CMPK2). CMPK2 is actively induced in response to several viral infections like hepatitis E virus (8), human immunodeficiency virus (9), dengue virus (DENV) (10), Nipah virus (11), and spring viremia of carp virus (12) and in response to lipopolysaccharide (LPS) and polyinosinic:polycytidylic acid (poly IC) (13) in macrophages. CMPK2, earlier believed to be a cytidine/uridine monophosphate kinase, was recently shown to be involved in the conversion of CDP/UDP to the respective triphosphates. CTP is then converted to ddhCTP by the adjacent gene RSAD2 (Viperin), which binds to nascent RNA and causes chain termination (14, 15). A recent study has demonstrated the involvement of the fish homolog 3nCmpk2 in antibacterial response by controlling bacterial colonization and protecting the intestinal barrier (16). In addition to its role in protective responses, CMPK2 has recently been implicated in activating host cell inflammasomes (17) by virtue of its role in mitochondrial DNA synthesis, thereby contributing to liver tissue injury and fostering premature atherosclerosis in systemic lupus erythematosus (18, 19).

This inherent duality of the inflammatory response—1) leading to infection control (host beneficial) and 2) extensive tissue damage on uncontrolled activation (detrimental)—signifies the need to effectively regulate the onset and end of the inflammation. Several mechanisms have been characterized for the control of inflammation by host phagocytes (20–23). Studies in the last decade have implicated a determinant connection between mitochondria and the inflammation balance of immune cells (24–26). The enormous metabolic flexibility offered by mitochondria resident processes forms the basis of cellular plasticity facilitating fine-tuned responses to physiological conditions (27, 28). The importance of mitochondrial health in terms of physiology, network architecture in immune cell function, and response to infection is well recognized (29–31). Activated M1 polarized macrophages demonstrate an important shift in the metabolic state relying completely on glycolysis for increased and faster production of energy (29, 32–35). This transition is associated with a distinctive change in mitochondrial structure and a diminished reliance on mitochondrial oxidative phosphorylation and increased production of mitochondrial reactive oxygen species (ROS) (36–39).

In line with our previous study on the robust induction of type I IFN response in Mtb-infected macrophages (40), we show that CMPK2, an ISG, is highly induced immediately following infection and is rapidly regulated to baseline levels in human macrophages. Altered expression of this gene is associated with a marked change in mitochondrial physiology indicating acute organelle stress; resting macrophages with constitutive silencing or overexpression of CMPK2 harbor significantly depolarized mitochondria with an atypical organelle network and heightened elaboration of mitochondrial ROS. This is reflected in the heightened levels of basal expression of inflammatory genes and a significant shift in macrophage metabolism to reliance on glycolysis resembling the M1 macrophages in these cells. Our study thus provides evidence for the importance of this gene in controlling inflammation in macrophages and the importance of regulated CMPK2 expression in protective responses, thereby highlighting an important link between mitochondrial metabolism and innate response kinetics of macrophages.

## Results

### Macrophages respond to infection/TLR4 stimulation by inducing CMPK2

**Figure 1:**
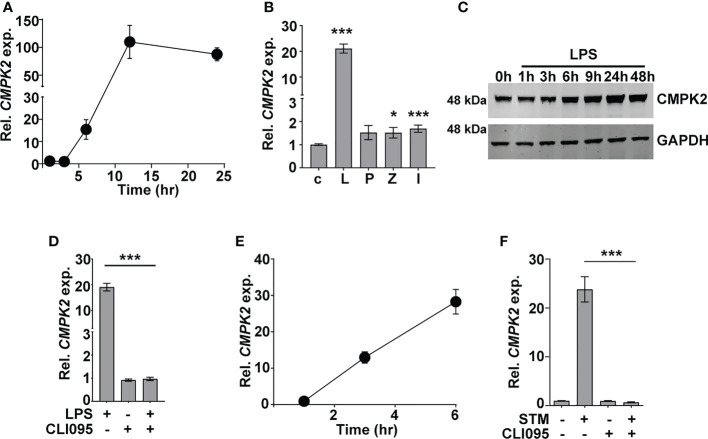
CMPK2 expression is induced following TLR4 stimulation of infection. **(A–F)** Analysis of CMPK2 expression in THP1 macrophages following: infection with Mtb at a MOI of 5 **(A)**, stimulation with various TLR ligands—c, untreated cells; L, LPS (10 ng/ml); P, Pam3CSK4 (20 ng/ml); Z, Zymosan (10 µg/ml); I, poly IC (2 µg/ml), **(B)** LPS stimulation by immunoblotting with CMPK2-specific antibody, **(C)** LPS stimulation with and without TLR4 inhibitor CLI095, **(D)** Infection with STM, **(E)** Infection with STM in the presence of CLI095, **(F)** Expression of *CMPK2* was quantitated by qPCR. Values are normalized with *GAPDH* and mean fold change compared to control cells ± SEM from N = 3 independent experiments. Mtb, *Mycobacterium tuberculosis*; MOI, multiplicity of infection; LPS, lipopolysaccharide; STM, *Salmonella enterica* subsp. enterica serovar Typhimurium. *p<0.05, ***p<0.001.

Given the early induction of type I IFN response in Mtb infections (5, 41, 42), we sought to investigate the importance of this pathway in the response dynamics of macrophages. The recent developments on the importance of mitochondrial physiology in host cell infection responses directed our attention to the mitochondria-associated ISG, *CMPK2*, which was highly induced following Mtb infection of macrophages. Infected THP1 macrophages showed a steady increase in *CMPK2* expression from 15-fold by 6 h and attaining peak levels of ~100-fold by 12 h and thereafter maintained even at 24 h of infection to greater than 80-fold (**Figure 1A**). In line with previous reports (17), we also observed a nearly 20-fold increase in *CMPK2* expression in response to LPS stimulation, while the other Toll-like receptor (TLR) ligands failed to alter this gene (**Figure 1B**). This was also evident as a steady increase in CMPK2 protein levels in THP1 macrophages even at 48 h of LPS stimulation (**Figure 1C**). The complete loss of LPS-induced expression in the presence of a TLR4 signaling inhibitor CLI095 confirmed the dependence of *CMPK2* expression on TLR4 signaling in macrophages (**Figure 1D**). Further, macrophages responded to *Salmonella enterica* subsp. *enterica* serovar Typhimurium (STM) infection by early induction of *CMPK2* expression with 12–13-fold increase by 3 h wherein they escalated further to ~28-fold by 6 h of infection (**Figure 1E**), and this response was completely abolished with the addition of CLI095, confirming the TLR4 engagement-dependent expression of this gene in macrophages (**Figure 1F**). A decrease in expression by more than 50% was observed in the case of macrophages infected with Mtb and treated with CLI095, Council of Scientific & Industrial Research again supporting the importance of TLR4 signaling in *CMPK2* expression (**Figure S1**).

### Modulation of CMPK2 activity affects the inflammation status of macrophages

**Figure 2:**
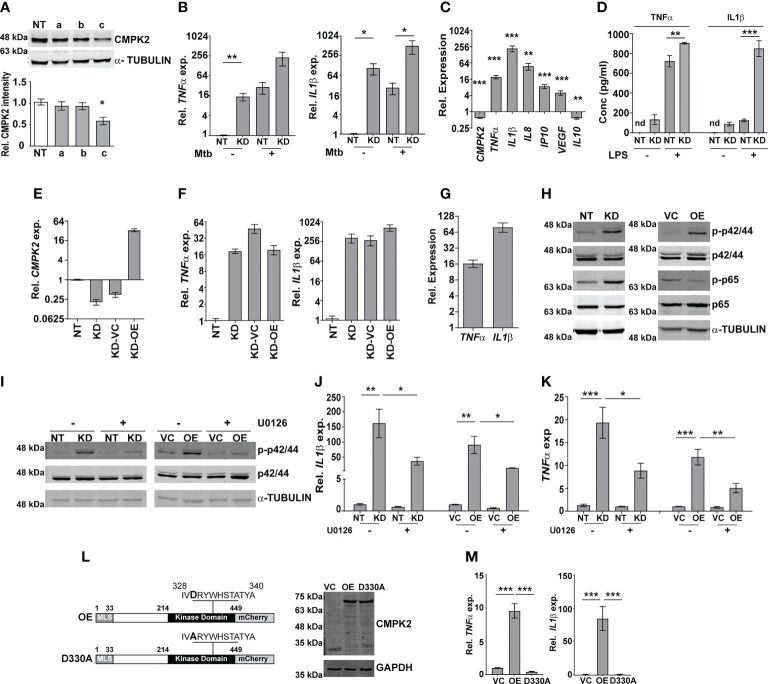
CMPK2 regulates the basal inflammation of macrophages. **(A)** Expression of CMPK2 in CMPK2 silenced macrophages by immunoblotting with specific antibodies. For immunoblotting, the expression of α-TUBULIN was used as control. One representative blot of three independent experiments is shown. The levels of CMPK2 expression are depicted as mean fold change ± SEM of N = 3 experiments by densitometry. **(B)** Expression of the *IL1β* and *TNFα* transcripts following infection with Mtb for 6 h was evaluated in NT or KD macrophages by qPCR. **(C)** Basal level expression of transcripts *CMPK2* and cytokine genes in KD macrophages was estimated by qPCR with specific primers. Values are normalized with *GAPDH* and mean fold change compared to control cells ± SEM from N = 3 independent experiments. **(D)** Levels of TNFα and IL1β in the culture supernatants of THP1 stably expressing non-targeting (NT) or siRNA specific to CMPK2 (KD) estimated by ELISA. Values are represented as mean pg/ml of the cytokine in triplicate assays from N = 3 experiments. **(E)** Expression of *CMPK2* in the CMPK2 silenced cells (KD) with stable expression of CMPK2 (KD-OE) or empty vector (KD-VC). The levels in the corresponding NT and KD cells are also shown. Values are represented as mean ± SEM of the triplicate assays from N = 3 experiments. **(F)** Expression of *TNFα* and *IL1β* in the CMPK2 silenced cells with stable expression of CMPK2 (KD-OE) or empty vector (KD-VC). The levels in the corresponding NT and KD cells are also shown. Values are represented as mean ± SEM of the triplicate assays from N = 3 experiments. **(G)** Expression of *TNFα* and *IL1β* in THP1 cells after stable expression of CMPK2. Values are represented as mean ± SEM of the triplicate assays from N = 3 experiments. **(H)** Analysis of activation of the ERK (p42/44) and NFκB (p65) signaling pathways in NT, KD, VC, and OE macrophages by immunoblotting with antibodies specific for the phosphorylated (active) and non-phosphorylated forms of the proteins. Representative blot of one experiment out of three individual assays is shown. Expression of α-TUBULIN was used as control. **(I)** ERK phosphorylation in macrophages with and without treatment with ERK specific inhibitor U0126. Antibodies specific for the phosphorylated (active) and non-phosphorylated forms of the proteins were used to probe cell extracts. Expression of α-TUBULIN was used as control. Blots are representative of two independent experiments. Expression of *IL1β* **(J)** and *TNFα* **(K)** in the four types of macrophages after treatment with U0126 was analyzed by qPCR. Cells left untreated were used as control. The relative gene expression fold in triplicate assay wells is represented with respect to *GAPDH* as mean ± SEM for N = 3. **(L)** The schematic of mutated catalytic site also depicted (D330A). Immunoblot analysis of CMPK2-mCherry fusion protein using an antibody specific for mCherry protein in protein lysates of VC, OE, and D330A THP1 cells with mCherry specific antibody. GAPDH was used as control. **(M)** Expression of *TNFα* and *IL1β* in THP1 cells after stable expression of vector alone (VC), full-length CMPK2 (OE), and catalytic mutant of CMPK2 (D330A). The relative gene expression fold in triplicate assay wells is represented with respect to *GAPDH* as mean ± SEM from N = 4. Mtb, *Mycobacterium tuberculosis*. *p<0.05, **p<0.01, ***p<0.001. nd, not detected.

To investigate the molecular function of CMPK2 in the macrophage response, we used *CMPK2*-specific siRNAs (a–c) to specifically silence this gene in THP1 cells (KD) and achieved a 60%–75% decrease in gene expression in comparison to non-targeting siRNA (NT) (**Figure S2A**). Only siRNA-c showed a significant decrease in protein levels in excess of 40% of the native CMPK2 expression and was used for further analysis (**Figure 2A**). Given the critical role of pro-inflammatory cytokines in the macrophage response to infection, we compared the levels of TNFα and IL1β in the NT and CMPK2 knockdown (KD) macrophages in response to Mtb infection. Mtb infection induced *TNFα* and *IL1β* expression by 200–500-fold in KD macrophages, while these levels were 10–20-fold lower in the case of NT macrophages (**Figure 2B**). Interestingly, expression levels of these genes were markedly elevated in the uninfected KD macrophages. This pattern was also reflected by the enhanced expression levels of other immune effectors like *IL8*, *IP10*, and *VEGF*, and a decrease in *IL10* in naïve KD macrophages strongly alluding to an enhanced pro-inflammatory status of these cells even in the resting state (**Figure 2C**). This profile of increased *TNFα* and *IL1β* gene expression was also observed in KD cells following LPS stimulation (**Figure S2B**). This enhanced expression also correlated well with the increase in secreted TNFα and IL1β in the culture supernatant of LPS-treated KD cells (**Figure 2D**). Again, correlating with gene expression, naïve KD cells also secreted increased amounts of both TNFα and IL1β in the supernatants as compared to the non-detectable levels of the cytokine in naïve NT macrophages.

Given the inflammatory phenotype observed with decreased CMPK2 expression in THP1 macrophages, we attempted to reverse this by complementing a functional copy of the gene in the silenced cells. We expressed the complete CMPK2 as a mCherry fusion protein in pCDNA3.1 and analyzed the basal expression levels of *IL1β* and *TNFα*. As expected, *CMPK2* expression was ~30-fold higher than in the empty vector control macrophages (**Figure 2E**). Contrary to our expectations, this enhanced expression of the *CMPK2* could not reverse the alteration in basal inflammation of these cells: nearly similar levels of *IL1β* and *TNFα* were observed in the complemented cells as in the silenced cells (**Figure 2F**). We hypothesized that similar to the situation with CMPK2 silenced cells, an increase in expression of CMPK2 was also detrimental to the inflammation homeostasis of macrophages. To test this, we analyzed the pro-inflammatory status of wild-type (Wt) THP1 cells stably expressing CMPK2-mCherry. Enhanced expression of *CMPK2* transcript in excess of 100-fold basal levels was observed in the overexpressing (OE) cells compared to the vector control (VC) cells (**Figure S2C**). The fusion protein of CMPK2 and mCherry was also clearly detected in protein extracts of the OE strains by immunoblotting (**Figure S2D**). Again, this enhanced expression of CMPK2 resulted in heightened levels of *TNFα* (12–15-fold) and *IL1β* (60–80-fold) in OE cells (**Figure 2G**).

In an effort to understand the basis for the hyper-inflammatory state of KD cells, we compared the immune signaling cascades of these cells with the NT cells in the basal state. Consistently, we observed enhanced activation of ERK and NFκB with 3-fold higher levels of phospho-p42/44 (ERK) and p65 NFκB (**Figures 2H**; **Figure S2E**) without any change in phospho-JNK/SAPK and p38 MAP kinase between the two cells (**Figure S2F**). This pattern of enhanced ERK phosphorylation was also observed in the case of OE cells in comparison to VC cells. The use of an ERK signaling specific inhibitor U0126 not only reduced the levels of phospho-p42/44 in both the KD and OE cells significantly (**Figure 2I**) but also significantly reduced the elevated levels of both *IL1β* and *TNFα* in both these cell types, further confirming the importance of ERK signaling in mediating the hyper-inflammatory phenotype of these macrophages (**Figures 2J, K**).

Human mitochondrial CMPK2 has been shown to be a nucleotide kinase involved in mitochondrial DNA synthesis (**Figure 2L**). In an effort to probe the importance of the kinase domain, we substituted the active site aspartate to alanine (D330A) as a kinase dead variant of CMPK2 (D330A) and analyzed the functional consequence of this expression on cytokine gene expression. Despite the strong and stable expression of D330A, comparable to the Wt, CMPK2 (OE), at both the transcript (**Figure S2G**) and protein (**Figure 2L**) levels, has was no evidence of increased basal inflammation. Contrasting with the significantly elevated levels of *TNFα* and *IL1β* in the OE cells (~10 and ~80 fold, respectively over basal levels), THP1 cells transfected with D330A harbored normal levels of gene expression (**Figure 2M**), emphasizing the importance of the kinase activity of CMPK2 in regulating basal inflammation in macrophages.

### Increased inflammation of knockdown macrophages is associated with altered mitochondria and augmented reactive oxygen species

**Figure 3:**
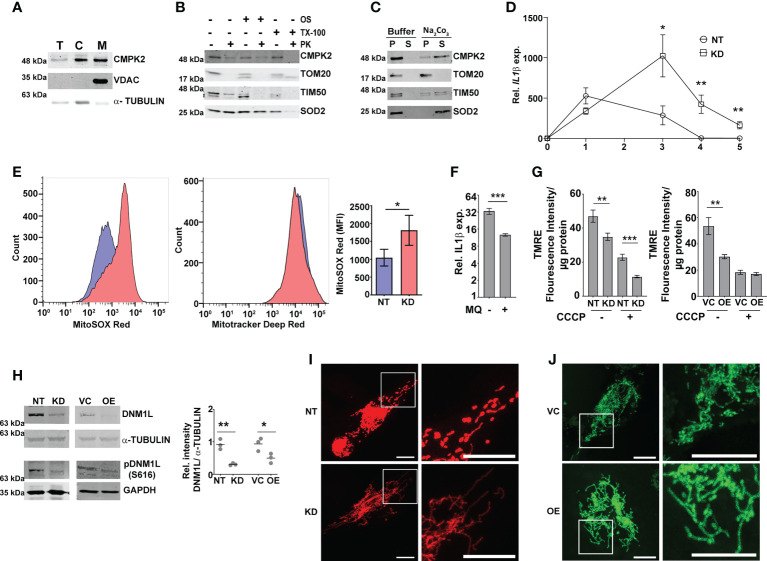
Modulation of CMPK2 affects the mitochondrial physiology in macrophages. **(A)** Expression of CMPK2 in subcellular fractions of THP1 macrophages by immunoblotting with specific antibodies: T, total cell extract; C, cytoplasmic fraction; M, mitochondria. Expression of cytosolic protein α-TUBULIN and mitochondria resident protein VDAC is also represented. Blots are representative of three independent experiments. **(B)** Mitochondrial fraction was subjected to Proteinase K treatment in the presence or absence of Triton X-100 and given osmotic shock (OS) and analyzed by immunoblotting. Data are representative of three independent experiments. **(C)** Mitochondrial fraction was incubated with mitochondrial buffer with and without Na_2_CO_3_ and centrifuged at 13,000 rpm for 15 min. The pellet (P) and supernatant (S) fractions were immunoblotted. Data are representative of three independent experiments. **(D)** Kinetic profile of IL1β expression in NT and KD during differentiation of monocytes to macrophages. Expression was checked at different time intervals after PMA treatment. Values are mean fold change in expression with respect to GAPDH + SEM triplicate assays of N = 3 experiments. **(E)** Analysis of mitochondrial ROS in NT or KD macrophages. Cells were stained with MitoTracker Deep Red (as an internal control), and ROS-specific MitoSOX Red and specific populations were quantified by FACS. The histogram plots of a representative experiment of N = 4 are depicted. The extent of MitoSOX red mean fluorescence intensity (MFI) + SEM is represented graphically in the inset. **(F)** Expression of IL1β in the macrophages with the addition of a specific mitochondrial ROS inhibitor MQ (1 µM) on day 1 of PMA treatment was analyzed by qPCR. Values are mean fold change in expression with respect to GAPDH + SEM for triplicate assays of N = 3 experiments. **(G)** Analysis of mitochondrial membrane potential in NT, KD, VC, and OE macrophages by TMRE staining. Fluorescence values normalized to protein in samples are represented as mean fluorescent intensity + SEM for triplicate assays of N = 3 experiments. **(H)** Expression of total and phosphorylated (S616) DNM1L by immunoblotting with specific antibody was analyzed and is depicted along with the levels of α-TUBULIN as a control. Relative intensity values are depicted as mean+ SEM of N = 3. **(I, J)** Mitochondrial architecture in control (NT) and KD **(I)** or VC and OE **(J)** macrophages were analyzed by confocal microscopy. A representative image is depicted with the scale bar representing 10 µm, and the region is shown in higher magnification as depicted by the box. PMA, phorbol 12-myristate 13-acetate; ROS, reactive oxygen species; FACS, fluorescence-activated cell sorting. *p<0.05, **p<0.01, ***p<0.001.

We presumed that identifying the precise localization of CMPK2 would reveal clues to its molecular role in inflammation and mitochondrial membrane dynamics. We first validated the mitochondrial localization of CMPK2 by immunoblotting subcellular fractions. With previous reports indicative of the mitochondrial localization of CMPK2 (14) and our observation of a majority of the protein in the mitochondrial fraction (**Figure 3A**), we tested purified mitochondrial fractions to pinpoint its location by standard biochemical and immunoblotting analyses. While treatment with Proteinase K (removes the surface exposed proteins) completely removed the outer membrane protein TOM20, nearly 30% of CMPK2 was present in the pellet fraction of intact mitochondria, similar to that observed for the other mitochondrial membrane-associated protein TIM50 (**Figure 3B**), suggesting its association with the outer membrane of mitochondria. Most of the matrix protein SOD2 remained in the pellet and was lost only with complete solubilization with Triton X-100. The peripheral association of CMPK2 with the mitochondrial membrane was also revealed by a higher amount of CMPK2 in the soluble fraction of mitochondria treated with Na_2_CO_3_ in contrast with the membrane-associated protein TOM20, which was not observed in the soluble fraction and remained in the pellet (**Figure 3C**). We hypothesized that two distinct possibilities could account for the enhanced basal levels of inflammation observed in the CMPK2 expression altered cells (KD and OE): 1) enhanced expression at all times or 2) a temporal activation of the signaling with delayed decay kinetics. To test this, we checked the expression of *IL1β* in the two cell lines during the monocyte-to-macrophage transition upon phorbol 12-myristate 13-acetate (PMA) treatment. While the monocytes did not show any difference in *IL1β* expression, activation with PMA induced distinct profiles of *IL1β* activation in the control (NT) or CMPK2 silenced (KD) cells. A rapid increase in *IL1β* within a day of activation (~500 fold) was followed by a steady decline by day 3 of treatment, further reaching basal levels by day 4 in the control cells (**Figure 3D**). CMPK2 silenced macrophages, however, displayed protracted response kinetics with a slower rise in expression levels ~1.5–2-fold lower than those of control cells at 24 h of activation with PMA. Between 24 and 72 h of treatment, the levels were 3–5-fold higher in KD with a delayed decline of the levels to ~400-fold by day 4 and did not fall to basal levels even by day 5.

Macrophage inflammation is strongly associated with ROS activation. To check this, we compared the levels of mitochondrial ROS in NT and KD macrophages by using a ROS-specific stain MitoSOX Red. MitoTracker deep red was used as the control for the levels of mitochondria in the two cell types. While similar levels of MitoTracker deep red staining hinted at unaltered mitochondrial levels, the level of ROS (MitoSOX) was nearly 1.5-fold higher in KD cells as compared to control NT macrophages (**Figure 3E**). The importance of mito-ROS in enhanced inflammation in KD cells was further evident when the cells were treated with a specific inhibitor mitoquinone (MQ). The addition of MQ early (immediately after PMA treatment) significantly reduced the expression of *IL1β* in these cells (**Figure 3F**); use of MQ at later stages of differentiation (48 and 72 h after PMA addition) did not alter the inflammation in the KD cells (**Figures S3A, B**). Mitochondrial membrane polarization is a critical component of organelle integrity and physiology with alterations in membrane potential leading to ROS in macrophages (43, 44). To test if the ROS was associated with a change in membrane potential, we compared the extent of TMRE staining (a dye that shows a potential dependent differential partitioning across mitochondrial membranes) in cells. As seen in **Figure 3G**, the mitochondria of both KD and OE macrophages exhibited significantly lower staining with TMRE, similar to the NT/VC cells treated with the proton pump uncoupler CCCP, suggestive of a loss of membrane integrity in the cells even at the basal level.

Given the importance of mitochondrial fission–fusion dynamics as a stress mitigating factor, we tested if the alteration in CMPK2 expression and the associated stress resulted in aberrant mitochondrial replication dynamics. In line with the previous report (45), both KD and OE cells showed decreased levels of total and phosphorylated (S616) dynamin-1-like protein (DNM1L), a critical component for mitochondrial fission in comparison to NT and VC cells, respectively (**Figure 3H**), contrasting with an unaltered expression of the mitochondrial fusion-associated proteins (MFN1 and OPA1) in these cells (**Figures S3C, D**). This was further evident in the larger and more dense mitochondrial network of these organelles in the KD and OE cells as compared to the small uniformly distributed mitochondria in the control cells (**Figures 3I, J**).

### Regulated levels of CMPK2 are critical for the normal metabolic activity of macrophages

**Figure 4:**
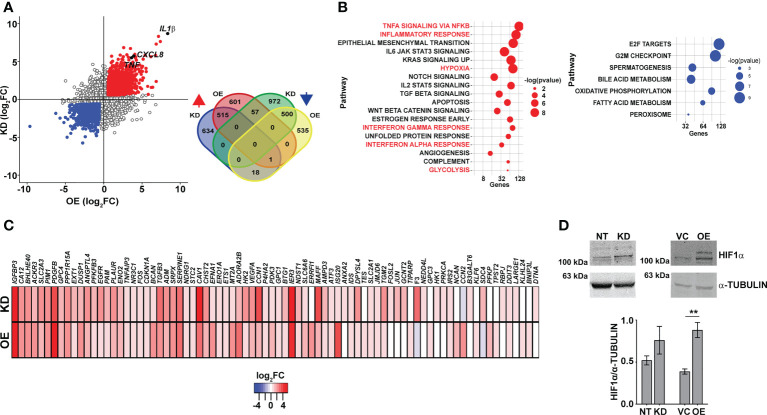
Macrophages with dysregulated CMPK2 display increased hypoxia. **(A)** Scatter plot of genes differentially expressed in KD or OE macrophages relative to the expression levels in the control macrophages (NT or VC), respectively. Change in expression is depicted as log_2_ fold change in expression. The number of genes upregulated and downregulated in the macrophages is depicted as a Venn diagram (inset). **(B)** GSEA hallmark pathway enrichment analysis of the commonly upregulated and downregulated genes in the KD and OE macrophages are represented as a bubble plot. X axis is the number of genes of the pathway and size of the bubble depicts significance (−log p-value). **(C)** Heat map of the hypoxia response gene expression in the CMPK2 dysregulated macrophages KD and OE. The values represent log_2_ fold change from the corresponding control cells. **(D)** Analysis of HIF1α in protein extracts of NT, KD, VC, and OE cells. Equal amounts of protein from the different extracts were probed with specific antibodies, and a representative blot of three independent experiments is depicted. The change in expression level of HIF1α with respect to α-TUBULIN was calculated by densitometric analysis and is graphically represented. GSEA, Gene Set Enrichment Analysis. **p<0.01.

In an attempt to understand the basis for this dysregulated inflammatory gene expression in both the KD and OE macrophages, we performed global sequencing of transcripts and compared the expression profiles with the control macrophages NT and VC. Strikingly, again, on the comparison of the differentially expressed genes, inflammatory cytokine genes like *IL1β*, *IL8*, and *TNFα* were commonly upregulated in both the KD and OE macrophages (**Figure 4A**). This consistency in gene expression profiles was also evident with the significantly large numbers of genes increased (515) or decreased (500) in macrophages with abnormal CMPK2 expression (**Figure 4A**, inset). Significant deregulation of basal level inflammation was also evident in Gene Set Enrichment Analysis (GSEA) with heightened inflammation response as a key gene family common to the two macrophage lines (**Figure 4B**). Interestingly, the hypoxia response was highlighted as one of the highly upregulated gene families observed in both the KD and OE macrophages. Most of the genes involved in the response were upregulated anywhere between 2- and 16-fold in these cells (**Figure 4C**). With hypoxia primarily regulated by HIF1α in cells, we probed the levels of this protein in extracts of these macrophages. HIF1α levels were elevated ~2-fold in the OE cells as compared to the control VC cells; similarly, a higher level of HIF1α was also observed in extracts of the KD cells (**Figure 4D**).

### Macrophages with dysregulated CMPK2 expression levels portray signatures of activated M1 cells

**Figure 5:**
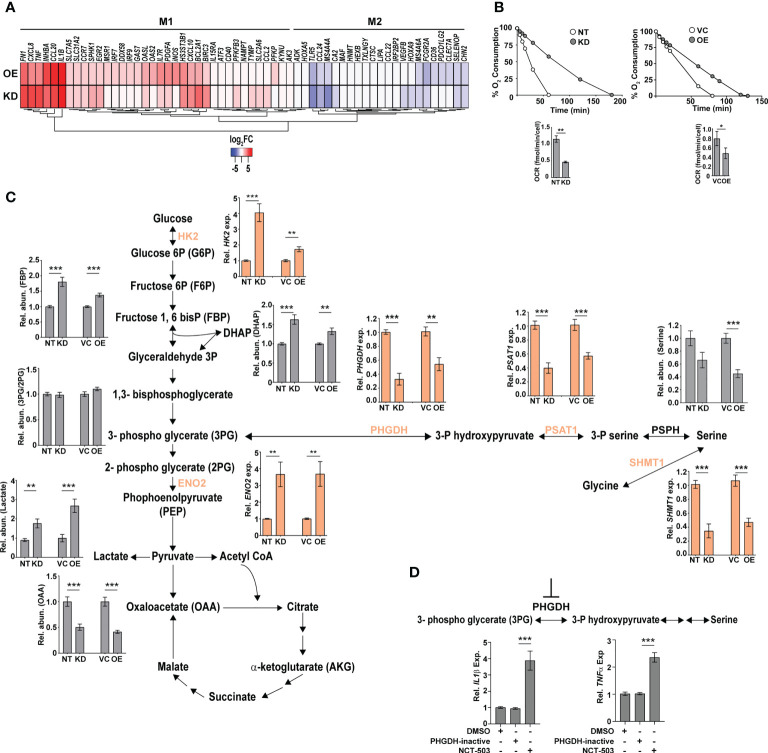
Macrophages with dysregulated CMPK2 are metabolically similar to activated M1 macrophages with higher glycolytic flux. **(A)** The expression patterns of genes specific to M1 and M2 activated macrophages in the CMPK2 dysregulated macrophages KD and OE are represented as heat maps. The values represent log_2_ fold change from the corresponding control cells. **(B)** Continual estimation of oxygen consumption in NT, KD, and VC, OE macrophages until 3 h with Oroboros oxygraph. The level of oxygen consumption was calculated, and mean OCR ± SEM of N = 3 experiments is shown. **(C)** Metabolite levels in the CMPK2 silenced (KD) and overexpression (OE) and their respective control macrophages were assayed from cellular extracts by MS. The levels of individual metabolites in the KD and OE macrophages are represented as relative abundance compared to control cells as mean ± SEM from three independent experiments (N = 3). Expression of genes involved in the glycolysis and serine-to-glycine biosynthetic pathway estimated by qRT-PCR is also shown. **(D)** Expression of *IL1β* and *TNFα* in THP1 cells treated with the serine biosynthesis inhibitor NCT-503 and its inactive form PHGDH-inactive. Expression values are mean fold change in expression with respect to *GAPDH* ± SEM triplicate assays of N = 3 experiments after 6 h of treatment. *p<0.05, **p<0.01, ***p<0.001.

The increased hypoxia, mitochondrial ROS, and inflammation observed in the KD and OE cells were reminiscent of M1-activated macrophages. To validate this, we analyzed the expression profiles of previously identified signatures of M1 and M2 polarized macrophages in our gene sets (46). In agreement with our hypothesis, both the KD and OE macrophages showed an increase in the expression of genes of M1 macrophages with a majority of the M2 specific signatures either unchanged or with reduced expression levels (**Figure 5A**). Further validation of an M1 bias was visible in the physiological state of the cells. Analysis of respiration rates of cells revealed a distinct pattern with a considerable decrease in the oxygen consumption rates of macrophages with altered expression of CMPK2. Both the KD and OE macrophages displayed 2–2.5-fold lower oxygen consumption with delayed kinetics as opposed to the control macrophages NT and VC (**Figure 5B**), implicating a strong shift of macrophages with altered CMPK2 expression toward a pro-inflammatory phenotype and the important role for CMPK2 in maintaining homeostasis of macrophages. Further, both these cell types harbored increased expression of genes involved in glycolysis (**Figure S4A**; **Figure 5C**) that manifested as a significant increase in the levels of glycolysis intermediates like fructose bis-phosphates, dihydroxy acetone phosphate (DHAP), culminating in markedly high levels of lactate (**Figure 5C**). Recent studies have demonstrated a strong link between serine biosynthesis/metabolism to the inflammatory profiles and mitochondrial physiology of cells (47–49). Strikingly, the serine–glycine biosynthetic pathway was one of the dominant metabolic axes downregulated in these two cell types in comparison with the control cells (**Figure S4B**). The expression levels of the key enzymes involved in this pathway were validated by qRT-PCR. Consistently, in both the KD and OE cells, expression levels of phosphoglycerate dehydrogenase (*PHGDH*), phosphoserine aminotransferase (*PSAT1*), and serine hydroxymethyltransferase1 (*SHMT1*) were significantly lower in comparison to those of the control NT and VC cells (**Figure 5C**). This decrease in expression levels is reflected as a ~2-fold decrease in the metabolite level of serine in the KD and OE cells. Given the important role of PHGDH in controlling inflammation under hypoxic conditions (50, 51) and our observation of a significant decrease in expression levels combined with hypoxic conditions and heightened pro-inflammatory gene expression in the KD and OE cells, we hypothesized an important role for this axis in the observed phenotypes in this study. To investigate this, we specifically inhibited PHGDH in THP1 Wt cells by the specific inhibitor NCT-503. As a control, cells were treated with an inactive inhibitor of PHGDH, and the expression profiles of *IL1β* and *TNFα* in these cells were evaluated. The use of the inactive inhibitor did not alter the expression from the untreated cells, in contrast to the significant increase in transcript levels by ~4- and 2.5-fold for these genes with the inhibitor, substantiating our supposition (**Figure 5D**).

### CMPK2 plays a critical role in the macrophage’s ability to control infection

**Figure 6:**
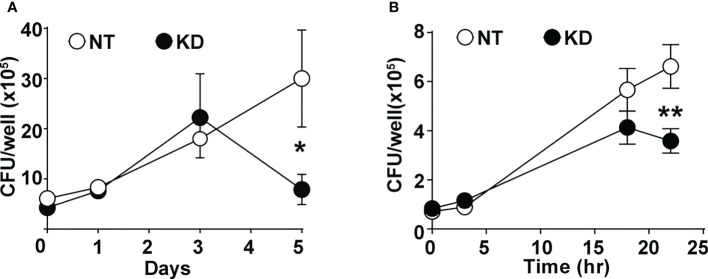
CMPK2 regulates the bactericidal activity of macrophages. **(A)** Growth kinetics of Mtb in NT and KD macrophages at different times post-infection at a MOI of 5 for 6 h Values are mean CFU ± SEM values in triplicate assays of N = 4. **(B)** Growth kinetics of STM in NT and KD macrophages at different times post-infection at a MOI of 10 for 20 min. Values are mean CFU ± SEM values in triplicate assays of N = 4. Mtb, *Mycobacterium tuberculosis*; MOI, multiplicity of infection; CFU, colony-forming unit; STM, *Salmonella enterica* subsp. enterica serovar Typhimurium. *p<0.05, **p<0.01.

To understand the impact of altered CMPK2 expression on macrophage inflammatory properties, we evaluated its ability to control the intracellular growth of Mtb. We observed a 2–3-fold decrease in intracellular bacterial numbers in these macrophages as compared to NT late in infection (day 5); KD cells were able to control bacterial levels to input levels by this timepoint compared to the growth of about 5–6-fold in the NT macrophages (**Figure 6A**). This pattern of increased bacterial control was also evident in STM-infected KD macrophages with 2–3-fold lower bacterial numbers after 24 h of infection (**Figure 6B**).

## Discussion

Being endowed with the capacity to initiate and manifest strong infection control programs, macrophages often dictate outcomes of infection as well as in the maintenance of tissue homeostasis during steady state and inflammation (52–56). Several studies have revealed the shift in macrophage physiology with a marked lowering of energy metabolism in macrophages by microbial infection (12, 57–59). A recent report has effectively implicated the infection-induced type I IFN response in lowering macrophage energy metabolism in Mtb infections (60). In fact, infection-dependent modulation of mitochondrial dynamics and function has been demonstrated as a key determinant of host cell response kinetics and immune signaling (38, 61–63).

We sought to investigate the mitochondria-associated gene CMPK2, also an IFN-induced gene, to decipher the importance of macrophage energy dynamics and metabolism in Mtb infections. The complete shift of macrophage physiology to uncontrolled inflammation by altered expression of CMPK2 argues for its critical role in regulating mitochondria integrity and macrophage physiology. Gene silencing (KD) as well as OE led to disruption of the normal mitochondrial physiology as evidenced by decreased membrane potential and increased ROS resulting in the enhanced activation of ERK signaling and a significant increase in the production of pro-inflammatory mediators like TNFα, IL8, and IL1β.

Proteins associated with the mitochondria require a carefully coordinated cytoplasmic transport into the various compartments of the organelle by a variety of mechanisms, and during failure, proteins tend to remain unfolded aggregates to be degraded by the host proteasome and non-functional (64, 65). Consistent with such studies, the removal of the mitochondrial localization signal (MLS) from CMPK2 destabilized the protein and resulted in enhanced degradation by the cellular proteolytic machinery. We also observed an absolute requirement of kinase activity for CMPK2 function and the importance of the N-terminal domain in protein stability (data not shown, as they are part of another manuscript).

Increased glycolytic flux with enhanced lactate production facilitates rapid energy generation for increased microbicidal capacity and is considered the hallmark of activated macrophages polarized to the M1 phenotype (66). Recent studies have also linked serine metabolism to physiology including inflammation in macrophages with an inflammation-promoting role for serine biosynthesis in macrophages (67, 68). Interestingly, inhibition of the key metabolic enzyme Phgdh and consequently serine synthesis resulted in enhanced expression of pro-inflammatory genes (50). Additionally, repression of serine catabolism by silencing SHMT2 in cancer cells has been shown to result in enhanced ROS under hypoxia (69). We demonstrate a significant shift in the metabolic status of CMPK2-modulated cells (KD and OE) toward rapid energy production *via* glycolysis and consequent accumulation of lactate in the cells. Moreover, enhanced ROS and hypoxia associated with a significant downregulation of genes involved in serine-glycine biosynthesis were also evident in these macrophages. The observed increase in the expression of pro-inflammatory genes in macrophages by specifically inhibiting PHDGH in this study in line with a previous study (50) only highlights another possible link between serine catabolism and cellular redox maintenance *via* the regulated expression of CMPK2.

Our observation of identical phenotypic characteristics of CMPK2 silenced as well as overexpression cells (enhanced mitochondrial stress even at the basal state) hinted at the possibility of a fundamental role of this gene in organelle function and integrity. Wang et al. effectively demonstrated that a change in the overall stoichiometric composition of larger protein complexes due to alteration in expression (increase or decrease) of SRPK1 resulted in similar phenotypes in cancerous cells (70). Our preliminary analysis of CMPK2 localizing with the mitochondrial membrane in proximity to the mitochondrial ETC proteins coupled with our observed decrease in the levels of complex 1 in CMPK2 dysregulated (KD and OE) macrophages is suggestive of the possible association of CMPK2 in large complexes (**Figure S5**). Moreover, the absolute requirement of kinase activity supports an important contribution of the purported activity of CMPK2 as a cytidine–uridine monophosphate kinase involved in the production of cellular CTP/UTP in the control of inflammation in macrophages in line with a previous study defining the importance of cellular pyrimidine levels in mitochondrial DNA-mediated regulated innate immunity (71). Comprehensive analysis toward pinpointing the precise molecular mechanism of CMPK2-mediated regulation of macrophage immuno-metabolism would help develop novel mechanisms of modulating innate response control, paving the way for future host cell-directed therapeutics.

Our data that a long-term breakdown of mitochondrial architecture and inflammation control in macrophages following prolonged disturbance of CMPK2 expression levels and the established importance of increased inflammation in activated macrophages lend credence to our hypothesis that CMPK2 is an important component of the cellular inflammation rheostat controlling macrophage metabolic and transcriptional response to infections.

## Materials and methods

### Reagents

The following chemicals were purchased: U0126 (U120), RNAzol RT (R4533), and Zymosan (Z4250) from Sigma-Aldrich (St. Louis, MO, USA); MitoSOX Red (M36008) and MitoTracker Deep Red (M22426) from Thermo Fisher Scientific (Waltham, MA, USA); and LPS (tlrl-smlps), CLI095 (tlrl-cli095), poly I:C (tlrl-pic), and Pam3CSK4 (tlrl-pms) from *In vivo*Gen (San Diego, CA, USA). Mitoquinol (89950), PHDGH-inactive (19717), and NCT-503 (19718) were purchased from Cayman Chemical Company (Ann Arbor, MI, USA).

### Cell culture

THP1 cells were cultured in RPMI-1640 (HiMedia Laboratories, Mumbai, India) media supplemented with 10% fetal bovine serum (FBS) and 1 mM of sodium pyruvate (HiMedia Laboratories, Mumbai, India). HEK293T cells were cultured in Dulbecco’s modified Eagle medium (DMEM) supplemented with 10% FBS and 1 mM of sodium pyruvate. For differentiation, THP1 cells were treated with 100 nM of PMA for 24 h and rested for 48 h before the next manipulation. The identity of both cell lines was confirmed by short tandem repeat (STR) PCR analysis, and the cell lines were maintained mycoplasma free by routine monitoring. LPS treatment of macrophages was performed with 10 ng/ml of LPS for 6 h. TLR4 signaling was inhibited by treatment with 10 µM of CLI095 before infection or LPS treatment. For ERK inhibition, THP1 macrophages were treated with 2 µM of U0126 for 6 h. To inhibit serine biosynthesis, macrophages were treated with 10 µM of NCT-503 for 6 h, and 10 µM of PHGDH-inactive compound was used as the negative control.

### Generation of THP1 lines with CMPK2 silencing

To generate knockdown cells, HEK293T cells were co-transfected with CMPK2 siRNA plasmid (Applied Biological Materials Inc., Richmond, BC, Canada), packaging plasmid (pCMV-dR8.2), and envelope plasmid (pCMV-VSV-G) using Lipofectamine-LTX reagent (Thermo Fisher Scientific India Pvt. Ltd., Mumbai, India). After 6 h of transfection, media was removed, and fresh media was added to the cells. After 48 h of transfection, the culture supernatant was collected and centrifuged at 500 rpm to remove cell debris. The virus particles in the supernatant were concentrated to 100 µl using Amicon^®^ Ultra-15 Centrifugal Filters (Sigma-Aldrich Chemicals Private Limited, Bengaluru, India) and used to infect THP1 cells. Cells were then selected with 0.6 µg/ml of puromycin, and green fluorescent protein (GFP)-positive clones were used for further studies.

### Bacterial culturing and infection

*M. tuberculosis* strain Erdman was grown in 7H9 Middlebrook media (BD Biosciences, San Jose, CA, USA) supplemented with Middlebrook ADC (BD, USA) at 37°C. *Escherichia coli* and *S. enterica* serovar Typhimurium were grown in Luria–Bertani (LB) media (HiMedia Laboratories, Mumbai, India) at 37°C. Single-cell suspension of mid-log phase bacteria was adjusted to the required cell density and then used to infect the differentiated THP1 macrophages at a multiplicity of infection (MOI) of 5. After 6 h *p.i.* for Mtb, cells were washed with phosphate-buffered saline (PBS) to remove extracellular bacteria, and at various times post-infection, bacterial numbers were enumerated by lysis of cells and plating for colony-forming unit (CFU) in 7H10 agar plates. For *S. typhimurium*/*E. coli*, post-addition of bacteria at a MOI of 10, the plates were centrifuged for 5 min and then left for 20 min at 37°C. The media was removed and treated with 100 μg/ml of gentamicin for 2 h in RPMI at 37°C. The cells were washed with PBS three times, and infection was continued in media containing 12 μg/ml of gentamicin, and at indicated time points, cells were lysed with PBS–Triton X-100 (1%) for CFU plating on LB agar (HiMedia Laboratories, Mumbai, India) plates. The colonies were counted and represented as CFU/well.

### Gene expression analysis by qPCR

Total RNA was isolated using RNAzol (Sigma-Aldrich, USA) method, and the concentration was quantified using NanoDrop 2000 UV–visible spectrophotometer. cDNA was prepared with 250–1,000 ng of total RNA by an RT-PCR using a Verso cDNA synthesis kit (Thermo Fisher Scientific, USA). qPCR was performed on a Roche LC480 II system using DyNAmo Flash SYBR Green mix (Thermo Fisher Scientific, USA). Primer sequences are given in **Table 1**.

**Table 1:** List of primers used in this study.

Name	Primer sequence
CMPK2 F	CCAGGTTGTTGCCATCGAAG
CMPK2 R	CAAGAGGGTGGTGACTTTAAGAG
IL8 F	AGACAGCAGAGCACACAAGC
IL8 R	ATGGTTCCTTCCGGTGGT
GAPDH F	GAAGGTGAAGGTCGGAGTC
GAPDH R	GAAGATGGTGATGGGATTTC
IL1β F	CCTGTCCTGCGTGTTGAAAGA
IL1β R	GGGAACTGGGCAGACTCAAA
TNFα F	CCCCAGGGACCTCTCTCTAATC
TNFα R	GGTTTGCTACAACATGGGCTACA
IP10 F	TCCACGTGTTGAGATCATTGC
IP10 R	GGCCTTCGATTCTGGATTCAG
IL10 F	GCTGGAGGACTTTAAGGGTTACCT
IL10 R	CTTGATGTCTGGGTCTTGGTTCT
VEGFα F	CTGCTGTCTTGGGTGCATTG
VEGFα R	CCATGAACTTCACCACTTCG
PP1	ATGGTACCATGGCCTTCGCCCGCCGGCTC
PP2	CGTCTAGAGGATCCGCCGGTTCACTAAAACTATTCTGG
PP3	CAAATCTCCTGTGATTGTAGCCAGGTACTGGCACAGCAC
PP4	GTGCTGTGCCAGTACCTGGCTACAATCACAGGAGATTTG

### Cloning of CMPK2 and the D330 variant

The NFκB promoter from pNFkB-d2GFP was excised by digestion with *Ecl*136II and *Hin*dIII, and the mCherry fragment from pmCherry-N1 was removed with *Hin*dIII and *Hpa*I and ligated to *Nru*I and *Eco*RV digested pcDNA3.1(+) to obtain the plasmid pPRAM1. The NFκB promoter from pPRAM1 was replaced with EF-1α from pTracer-EF/V5 His A by using *Mlu*I and *Eco*RI to obtain pPRAM2. The CMPK2 fragments were amplified from THP1 cDNA using the primer sets PP1 and PP2 and cloned into the pPRAM2 using *Kpn*I and *Bam*HI. The D330A catalytic site mutant was prepared by site-directed mutagenesis using primers PP3 and PP4. The sequences were verified by sequencing. The list of primers used in this study is given in **Table 1**.

### Western blotting and ELISA

For immunoblotting, cells were lysed in radioimmunoprecipitation assay (RIPA) buffer, resolved by sodium dodecyl sulfate–polyacrylamide gel electrophoresis (SDS-PAGE), and transferred to a nitrocellulose membrane. After being blocked with 5% bovine serum albumin (BSA) in Tris-Buffered Saline Tween (TBST) for 1 h at room temperature, membranes were incubated with the appropriate dilution of the primary antibody overnight, washed with PBS and probed with infrared dye conjugated secondary antibody, and developed in the LI-COR Odyssey platform. The following antibodies were purchased: rabbit anti-α-tubulin (2125), rabbit anti-phospho-p42/44 (4377), rabbit anti-p42/44 (4695), rabbit anti-phospho-NFkB p65 (3033), rabbit anti-NFkB p65 (4764), rabbit anti-phospho-JNK (4671), and rabbit anti-JNK (9258) from Cell Signaling Technology; rabbit anti-mCherry (ab167453), mouse anti-TOM20 (ab56783), rabbit anti-TIM50 (ab109436), rabbit anti-MFN1 (ab104585), mouse anti-OPA1 (ab194830), rabbit anti-DNM1L (ab140494), rabbit anti-p-DNM1L (S616) (4494S), and rabbit anti-HIF1α (ab51608) from Abcam; rabbit anti-VDAC (D73D12) and mouse anti-SOD2 (611581) from BD Biosciences; and gat anti-rabbit IgG 800 (926-32211) and goat anti-mouse IgG 680 (926-68070) from LI-COR Biotechnology. Rabbit anti-CMPK2 (HPA041430) was purchased from Sigma-Aldrich.

For ELISA, either cell supernatants or cell extracts were used for cytokine estimation with specific kits—TNFα (cat no. 88-7346-77, eBioscience) and IL1β (cat no 557953, BD Biosciences) ELISA kits—according to the manufacturers’ protocol.

### Analysis of reactive oxygen species by fluorescence-activated cell sorting

THP1 monocyte-derived macrophages (MDMs) were removed with PBS and 4 mM of EDTA, washed with PBS, resuspended in RPMI media, and used for staining with 5 µM of MitoSOX Red and 100 nM of MitoTracker Deep Red at 37°C for 30 min. Cells were then washed twice in Hanks’ Balanced Salt Solution (HBSS), finally resuspended in 500 µl of HBSS, and analyzed in FACS ARIA II (BD Biosciences).

### Metabolite measurement

Intracellular metabolites for MS-based targeted metabolomics were extracted using methanol–water. Briefly, 1.2 × 10^6^ cells from each condition were washed three times with ice-cold PBS and then quenched with methanol–water (4:1). The cell suspension was freeze-thawed in liquid nitrogen three times. The suspension was centrifuged at 15,000 *g* at 4°C for 10 min. The supernatant was collected, vacuum-dried, and then reconstituted in 50 μl of 50% methanol. The reconstituted mixture was centrifuged at 15,000 *g* for 10 min, and 5 μl was injected for Liquid chromatography–tandem mass spectrometry (LC–MS/MS) analysis.

The data were acquired using a Sciex Exion LCTM analytical ultra-high performance liquid chromatography (UHPLC) system coupled with a triple quadrupole hybrid ion trap mass spectrometer (QTrap 6500; Sciex, Framingham, MA, USA) in negative ion mode. Samples were loaded onto an Acquity UPLC BEH HILIC (1.7 μm, 2.1 × 100 mm) column, with a flow rate of 0.3 ml/min. The mobile phases were composed of 10 mM of ammonium acetate and 0.1% formic acid (buffer A) and 95% acetonitrile with 5 mM of ammonium acetate and 0.1% formic acid (buffer B). The linear mobile phase was applied from 95% to 20% of buffer A. The gradient program was used as follows: 95% buffer B for 1.5 min, 80%–50% buffer B in the next 0.5 min, followed by 50% buffer B for the next 2 min, and then decreased to 20% buffer B in the next 50 s, 20% buffer B for the next 2 min, and finally again 95% buffer B for the next 2 min. After data acquisition, peaks corresponding to each metabolite were extracted using Sciex Multiquant TM v.3.0 software, and the area was exported in an Excel sheet. Normalization was performed using the total area sum of that particular run.

### Oxygen consumption

To the chambers of the Oxygraphy-2k (O2k, Oroboros Instruments, Innsbruck, Austria), 3–4 × 10^6^ cells of THP1 MDMs in complete RPMI were added. The oxygen concentration was measured over time at 37°C under constant stirring. The oxygen consumption rate was calculated using the XY graph of oxygen consumption and time.

### Transcriptome analysis

Total RNA was isolated using RNAzol (Sigma Aldrich, India), and transcript sequencing was performed commercially by Bencos Research Solutions Pvt. Ltd. (Bengaluru, India). cDNA libraries were generated using Truseq RNA Library Prep Kit (Illumina, San Diego, CA, USA) and sequenced in an Illumina Novaseq 6000 platform. RNA-seq reads were aligned with the hg38 genome using STAR (72). HTSeq-count was used to count the transcripts (73). The differential expression analysis across samples was analyzed using GSEA (74).

### Confocal microscopy and image analysis of stained cells

THP1 monocytes (stable transfectants of non-targeting or CMPK2 specific siRNA) were transfected with plasmid DNA mtDsRed (kind gift by Dr. Soumya Sinha Roy, CSIR-IGIB) and selected for stably expressing clones with 400 µg/ml of G418. Cells were differentiated on coverslips, fixed with 4% formaldehyde, and imaged in a Leica 480 confocal microscopy (Leica Biosystems, Wetzlar, Germany). For live imaging, monocytes overexpressing CMPK2 were plated at a cell density of 0.3 × 10^6^ cells/ml in the cell culture dish containing two chambers per dish (1 ml/chamber). MitoTracker Green dye was added to the media, and the cells were kept in a 37°C CO_2_ incubator for 20 mins. The cells were imaged on a Leica SP8 confocal microscope.

### Mitochondrial localization

Differential centrifugation was employed for mitochondria isolation from the cells (75). Briefly, 2.5 × 10^6^ of THP1 MDMs were washed twice with PBS and resuspended in 3 ml of ice-cold cell isolation buffer (IBc-containing 10 mM of Tris, pH to 7.4, 1 mM of EGTA pH to 7.4, and 0.2 M of sucrose). The cells were homogenized using a glass Teflon pestle potter. The homogenate was then centrifuged at 600 g for 10 min at 4°C; the supernatant was collected and centrifuged at 7,000 *g* for 10 min at 4°C. The pellet was then washed, resuspended in 200 µl of IB_C_, and transferred to 1.5-ml Eppendorf tubes. The homogenate was again centrifuged at 7,000 *g* for 10 min at 4°C. The supernatant was discarded, and the pellet was resuspended to obtain the mitochondrial fraction.

For Proteinase K digestion, the mitochondria were resuspended in MS buffer (210 mM of mannitol, 70 mM of sucrose, 5 mM of Tris-HCL, pH 7.5, 1 mM of EDTA, pH 7.6/20 mM HEPES with or without 2 mg/ml of Proteinase K (for 15 min) and/or 1% Triton X-100. The reaction was terminated by the addition of 5 mM of phenylmethylsulfonyl fluoride. The mixture was centrifuged at 15,000 *g* for 15 min, and the pellet fraction was collected. For analysis of membrane proteins, the mitochondrial fraction was resuspended in MS buffer with and without 0.1 M of Na_2_CO_3_ and incubated in ice for 30 min. The pellet and supernatant fractions were collected at 15,000 *g* for 15 min. All fractions were analyzed by immunoblotting with respective antibodies.

### Graphs and statistical analysis

Statistical analyses were performed by using the two-tailed Student’s t-test. GraphPad Prism software was used for graphs and statistical analysis.

## Data availability statement

The datasets presented in this study can be found in online repositories. The names of the repository/repositories and accession number(s) can be found below: https://www.ncbi.nlm.nih.gov/, GSE199951.

## Author contributions

PA, SR, SG, and VR were involved in conceptualizing and design of the work. Experiments were performed by PA, TR, MC, KB, MM, and DS. PA and RC performed the mass spectrometry. PA, SG, and VR wrote the manuscript. All authors contributed to the article and approved the submitted version.

## Funding

The authors thank CSIR (VR-BSC0123, MLP2012), and DBT (SG-GAP0088) funding agency for supporting the study. The authors thank CSIR- BSC0403 for the confocal microscopy facility and CSIR-STS0016 for BSL3 facility.

## Acknowledgments

The authors thank Mr. Manish Kumar for the confocal microscopy facility and CSIR-IGIB for the BSL3 facility. The authors wish to thank Dr. Soumya Sinha Roy, CSIR-IGIB, for the plasmid pmtDsRed. The authors thank PA’s doctoral advisory committee members for useful discussions and comments on the project. The mass spectrometry facilities are duly acknowledged. The student fellowships were from CSIR: PA-CSIR-SRF, RA, PA-CSIR-BSC0124, MC-CSIR-JRF, India. The authors thank Nikita Bhor for designing the graphical abstract of this manuscript.

## Conflict of interest

The authors declare that the research was conducted in the absence of any commercial or financial relationships that could be construed as a potential conflict of interest.

## Publisher’s note

All claims expressed in this article are solely those of the authors and do not necessarily represent those of their affiliated organizations, or those of the publisher, the editors and the reviewers. Any product that may be evaluated in this article, or claim that may be made by its manufacturer, is not guaranteed or endorsed by the publisher.

## Supplementary material

Supplementary Figure 1 Expression of *CMPK2* in THP1 macrophages upon Mtb infection in the presence or absence of CLI095.

Supplementary Figure 2
**(A)** Expression of *CMPK2* in THP1 macrophages stably expressing the non-targeting (NT) siRNA or three different siRNAs **(a-c)** against CMPK2 by qPCR. The gene expression was normalized with *GAPDH* and relative fold changes compared to NT are represented as mean ± SEM (N=3). **(B)** Expression of *TNFα* and *IL1β* gene in NT and KD macrophages following LPS stimulation for 6 h. The relative gene expression folds in triplicate assay wells are represented with respect to *GAPDH* as mean ± SEM for N=3. **(C)** Expression of *CMPK2* in OE or empty vector -VC cells was analyzed by qPCR. The relative gene expression folds in triplicate assay wells are represented with respect to *GAPDH* as mean ± SEM for N=3. **(D)** CMPK2 expression in VC and OE cells was analyzed by immunoblotting with tag specific (mCherry) antibody. **(E)** Relative quantitation of p-ERK and p-NFκB in the NT, KD, VC, OE cells by densitometric analysis of immunoreactivity is shown. Values are mean + SEM of triplicate (N=3) independent blots. **(F)** Analysis of activation of the p38 MAPK and SAPK/JNK signaling pathways in NT and KD macrophages by immunoblotting with antibodies specific for the phosphorylated (active) and non- phosphorylated forms of the proteins. The relative intensities of the blots in the CMPK2 silenced cells w.r.t control (NT) cells are represented as mean ± SEM of N=3. Expression of α-TUBULIN was used as control. **(G)** Expression of *CMPK2* in VC, OE and D330A cells was analyzed by qPCR and is depicted relative to GAPDH levels of N=3 assays.

Supplementary Figure 3
**(A, B)** Expression of *IL1β* in the macrophages with the addition of a specific mitochondrial ROS inhibitor- MQ at day 2 [A] and day 3 [B] of PMA treatment intervals post activation was analyzed by qPCR. Values are mean fold change in expression with respect to *GAPDH* ± SEM for triplicate assays of N=3 experiments. **(C, D)** Expression of proteins involved in mitochondrial fusion by immunoblotting with specific antibodies. MFN1&2 and OPA1 were checked in NT, KD **(C)** or VC, OE **(D)** cells along with α-TUBULIN levels as a control. Relative intensity values are depicted ± SEM of N=3.

Supplementary Figure 4
**(A)** Expression of genes involved in glycolysis and TCA was represented as heatmap. The expression was extracted from the RNA sequencing data of CMPK2 dysregulated THP1 cells. The values are represented as log_2_ compared to respective control cells. **(B)** Panther gene family enrichment analysis of the commonly up and down regulated genes in the KD and OE macrophages is represented as a bubble plot. X axis is the number of genes of the pathway.

Supplementary Figure 5 Analysis of expression of protein complexes of the mitochondrial respiratory chain in cell lysates of NT and KD macrophages by immunoblotting. α-TUBULIN (T) and VDAC1 (VD) were used as loading controls. The band intensities of the complex proteins were normalized to α-TUBULIN and the relative intensity of KD w.r.t. NT is represented as fold change ± SEM for N=2.
