# Supplementary figures and images for: The mitochondrial gene-CMPK2 functions as a rheostat for macrophage homeostasis
Source: Front Immunol. 2022 Nov 14;13:935710. doi: 10.3389/fimmu.2022.935710 (PMC9702992; doi:10.3389/fimmu.2022.935710)

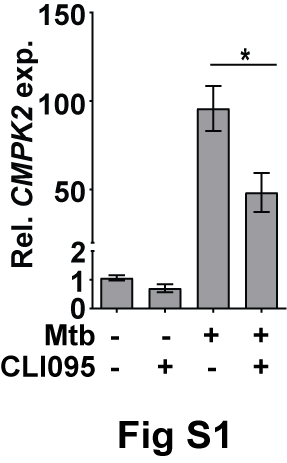

Supplement: Supplementary Figure 1 — Expression of CMPK2 in THP1 macrophages upon Mtb infection in the presence or absence of CLI095. [file Image_1.tif]

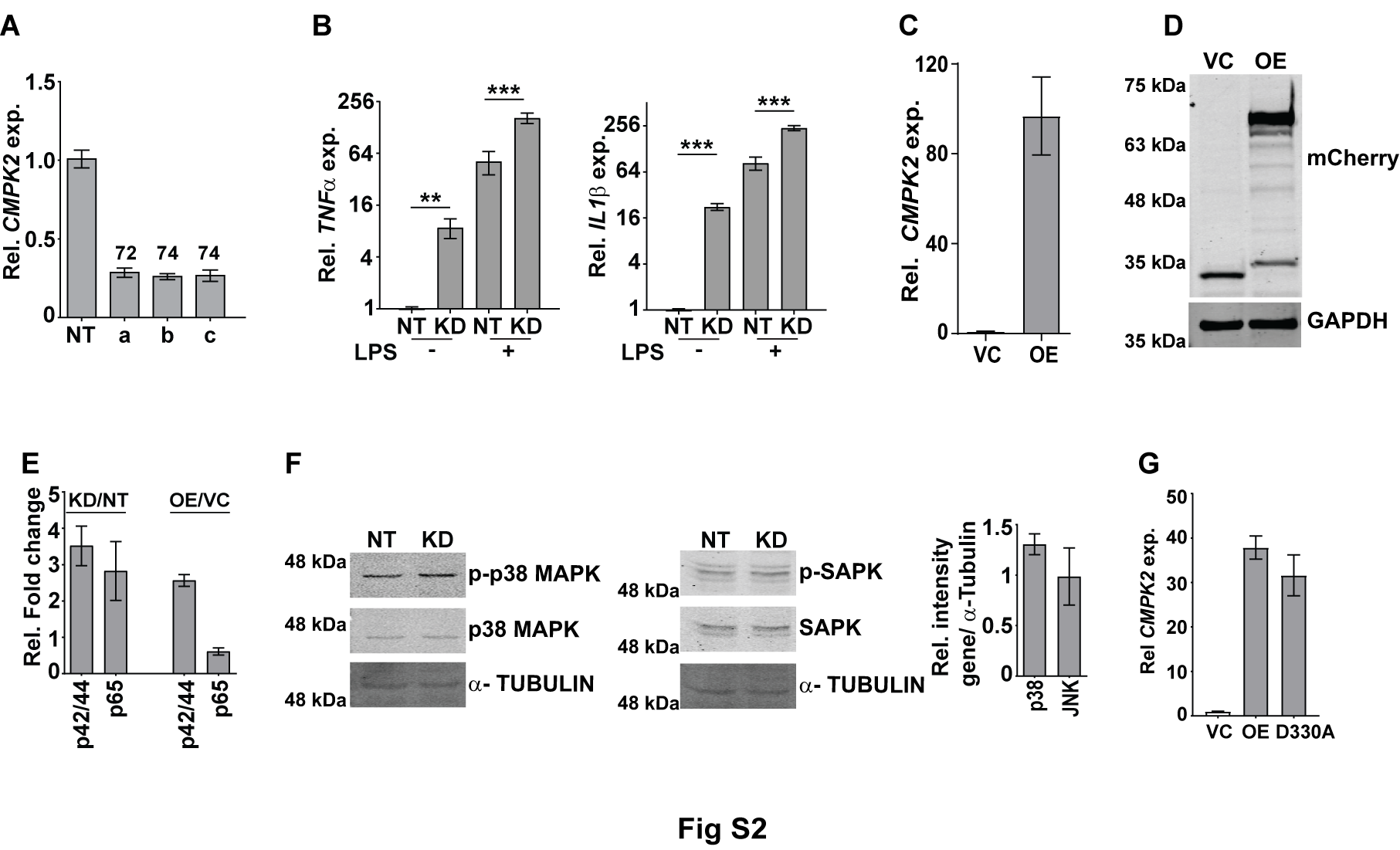

Supplement: Supplementary Figure 2 — (A) Expression of CMPK2 in THP1 macrophages stably expressing the non-targeting (NT) siRNA or three different siRNAs (a-c) against CMPK2 by qPCR. The gene expression was normalized with GAPDH and relative fold changes compared to NT are represented as mean ± SEM (N=3). (B) Expression of TNFα and IL1β gene in NT and KD macrophages following LPS stimulation for 6 h. The relative gene expression folds in triplicate assay wells are represented with respect to GAPDH as mean ± SEM for N=3. (C) Expression of CMPK2 in OE or empty vector -VC cells was analyzed by qPCR. The relative gene expression folds in triplicate assay wells are represented with respect to GAPDH as mean ± SEM for N=3. (D) CMPK2 expression in VC and OE cells was analyzed by immunoblotting with tag specific (mCherry) antibody. (E) Relative quantitation of p-ERK and p-NFκB in the NT, KD, VC, OE cells by densitometric analysis of immunoreactivity is shown. Values are mean + SEM of triplicate (N=3) independent blots. (F) Analysis of activation of the p38 MAPK and SAPK/JNK signaling pathways in NT and KD macrophages by immunoblotting with antibodies specific for the phosphorylated (active) and non- phosphorylated forms of the proteins. The relative intensities of the blots in the CMPK2 silenced cells w.r.t control (NT) cells are represented as mean ± SEM of N=3. Expression of α-TUBULIN was used as control. (G) Expression of CMPK2 in VC, OE and D330A cells was analyzed by qPCR and is depicted relative to GAPDH levels of N=3 assays. [file Image_2.tif]

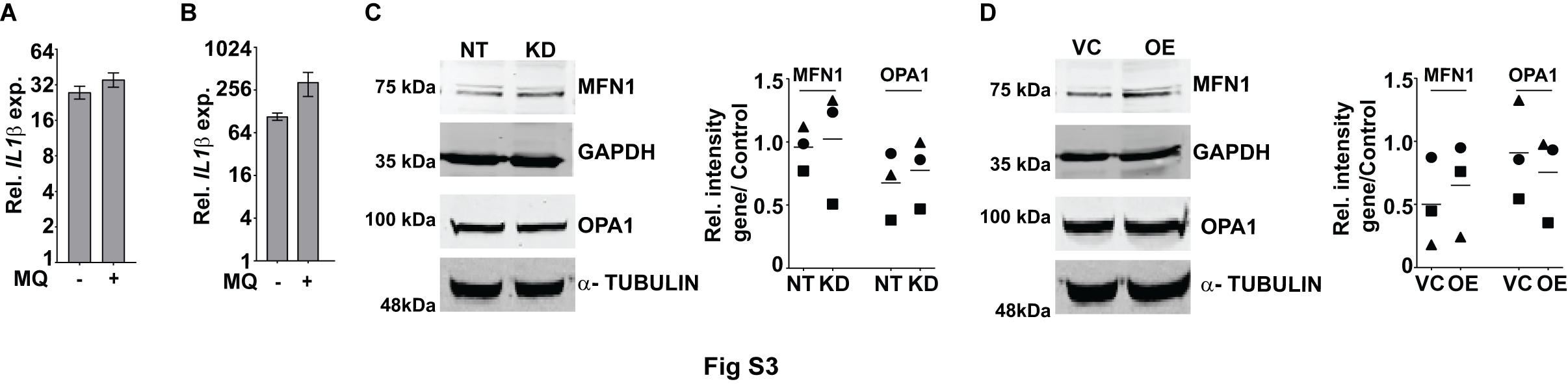

Supplement: Supplementary Figure 3 — (A, B) Expression of IL1β in the macrophages with the addition of a specific mitochondrial ROS inhibitor- MQ at day 2 [A] and day 3 [B] of PMA treatment intervals post activation was analyzed by qPCR. Values are mean fold change in expression with respect to GAPDH ± SEM for triplicate assays of N=3 experiments. (C, D) Expression of proteins involved in mitochondrial fusion by immunoblotting with specific antibodies. MFN1&2 and OPA1 were checked in NT, KD (C) or VC, OE (D) cells along with α-TUBULIN levels as a control. Relative intensity values are depicted ± SEM of N=3. [file Image_3.tif]

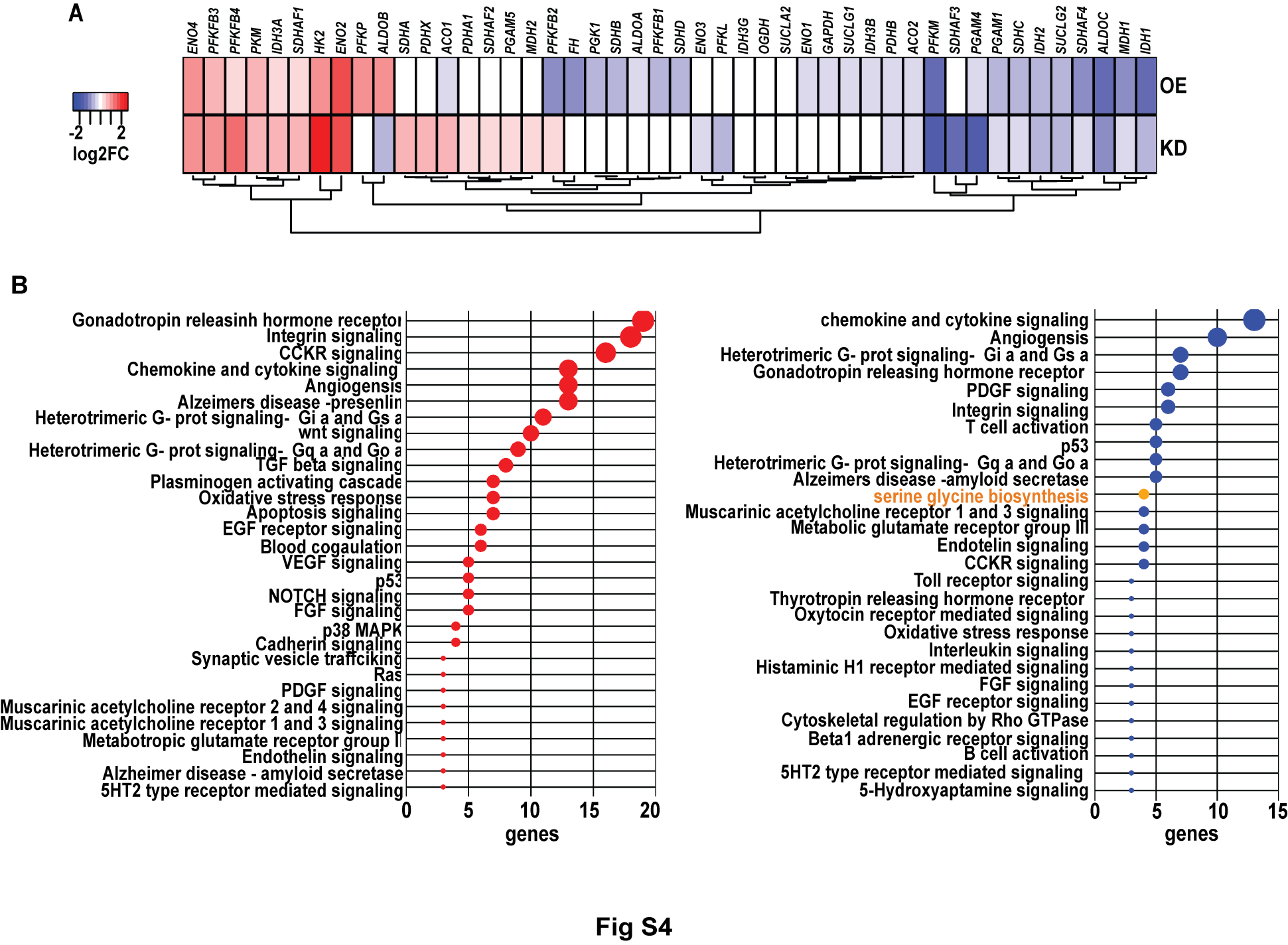

Supplement: Supplementary Figure 4 — (A) Expression of genes involved in glycolysis and TCA was represented as heatmap. The expression was extracted from the RNA sequencing data of CMPK2 dysregulated THP1 cells. The values are represented as log2 compared to respective control cells. (B) Panther gene family enrichment analysis of the commonly up and down regulated genes in the KD and OE macrophages is represented as a bubble plot. X axis is the number of genes of the pathway. [file Image_4.tif]

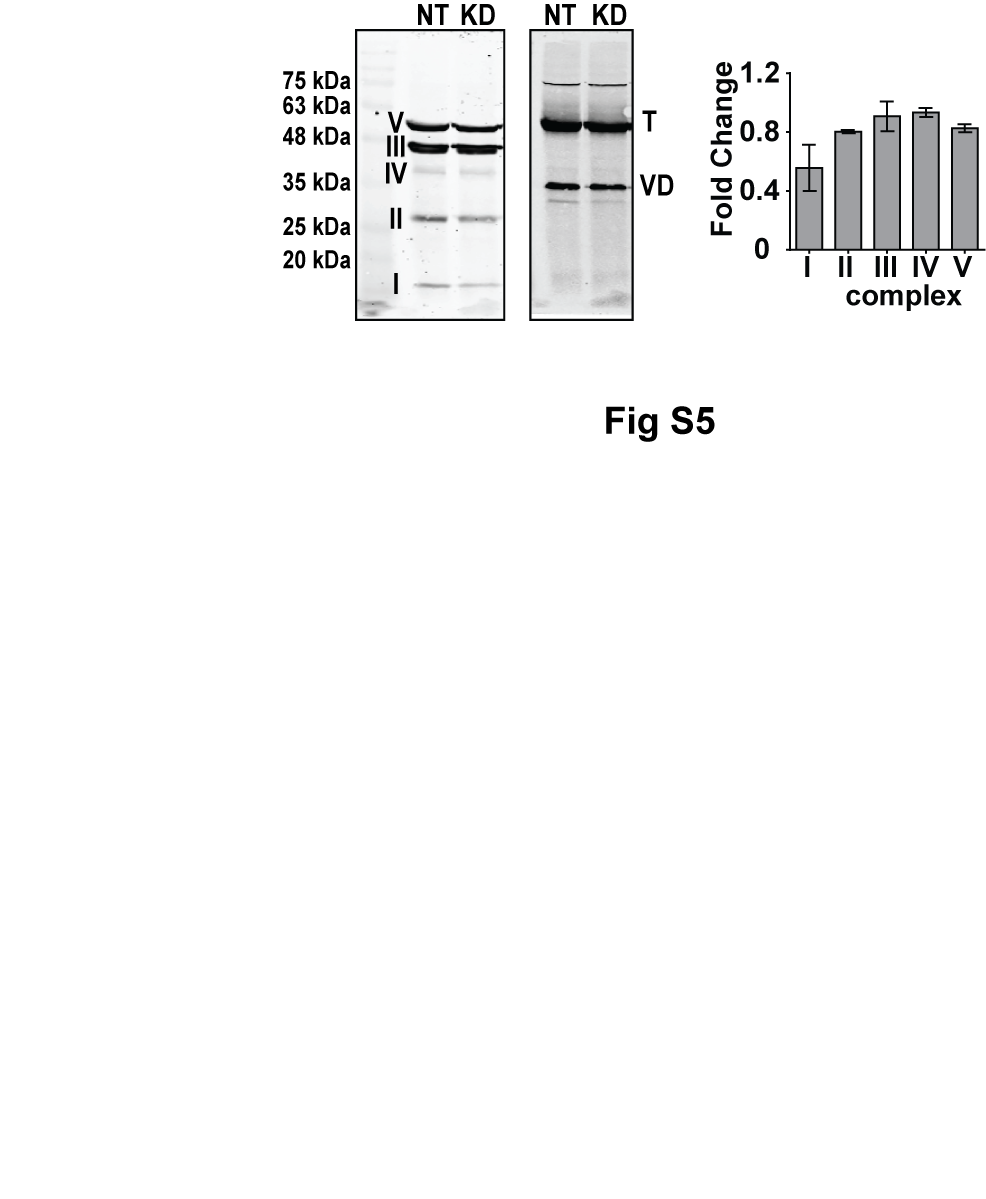

Supplement: Supplementary Figure 5 — Analysis of expression of protein complexes of the mitochondrial respiratory chain in cell lysates of NT and KD macrophages by immunoblotting. α-TUBULIN (T) and VDAC1 (VD) were used as loading controls. The band intensities of the complex proteins were normalized to α-TUBULIN and the relative intensity of KD w.r.t. NT is represented as fold change ± SEM for N=2. [file Image_5.tif]
